# New Cretaceous Fossil Achilidae Taxa (Insecta, Hemiptera, Fulgoromorpha) from Burmese Ambers with Description of Niryasaburniini Trib. Nov.

**DOI:** 10.3390/insects15040252

**Published:** 2024-04-07

**Authors:** Keyi Deng, Thierry Bourgoin, Zhiyue Zhai, Menglin Wang

**Affiliations:** 1 Key Laboratory of Southwest China Wildlife Resources Conservation, Ministry of Education, China West Normal University, Nanchong 637009, China; dky525129@163.com (K.D.); zzy050617zzy@163.com (Z.Z.); 2 College of Life Sciences, China West Normal University, Nanchong 637009, China; 3 Institut de Systématique, Evolution, Biodiversité, ISYEB-UMR 7205 MNHN-CNRS-Sorbonne Université-EPHE-University Antilles, Muséum National d’Histoire Naturelle, CP 50, 57 rue Cuvier, 75005 Paris, France; thierry.bourgoin@mnhn.fr

**Keywords:** planthopper, Cenomanian, Myanmar fossil, new taxa, morphology

## Abstract

**Simple Summary:**

An increasing number of fossil planthoppers from Burmese ambers are regularly newly described, yet few belong to extant families. In this study, we describe new fossil taxa in the family Achilidae: *Niryasaburnia nigrutomia* sp. nov. and *Sinuovenaxius kachinensis* gen. et sp. nov. Additionally, we propose the establishment of a new tribe, Niryasaburniini trib. nov., to accommodate these two genera. A key for identifying tribes within the Myconinae subfamily is provided.

**Abstract:**

A new species *Niryasaburnia nigrutomia* sp. nov. of the planthopper family Achilidae from Burmese amber collected from Hukawng Valley (Tanai) of northern Myanmar, is described, notably based on forewing pattern coloration and metatibiotarsal teeth conformation. A new fossil genus with its type species *Sinuovenaxius kachinensis* gen. et sp. nov. is also described. The tribe Niryasaburniini trib. nov. is established to include *Niryasaburnia* Szwedo, 2004, and *Sinuovenaxius* gen. nov., based on a unique combination of characters, of which the following states are particularly notable: head with compound eyes around half the length of pronotum, late forking of ScP+R and CuA after the fusion of Pcu+A_1_ on the forewing, apical teeth of metatarsomeres I and II both with subapical platellar sensilla, and a unique hindwing pattern with simple RP and biforked MP, CuA with two terminals only, and with A_2_ simple, reaching the posterior wing margin. The hindwing venation of this new tribe with RP with only one terminal and both MP and CuA with two terminals is unique in Achilidae.

## 1. Introduction

Recent studies place the origin of Achilidae Stål, 1866 in the Jurassic period [1], and this may even extend to the end of the Triassic [2,3]. By the mid-Cretaceous period, approximately 100 million years ago, all major lineages of Achilidae had emerged and began diversifying [2,3]. It is therefore not surprising to find a significant presence of Achilidae in Burmese amber inclusions, where they are the second most diverse of extant planthopper families in the Cretaceous fossil record, following Cixiidae Spinola, 1839, and surpassing Derbidae Spinola, 1839 and Nogodinidae Melichar, 1898 [4,5,6,7,8,9].

The taxon Achilidae was initially separated by Stål as the subfamily ‘Achilida’ within the family ‘Fulgorida‘ in his work “Hemiptera Africana” in 1866 [10]. The taxon was later formally recognized as a distinct family by Muir in 1923 [11]. The evolutionary history and taxonomic divisions within Achilidae have been reviewed by Brysz and Szwedo [12].

The Achilidae currently group 162 genera and 521 species, respectively only accounting for 6.4% and 3.7% of Fulgoromorpha diversity [13]. Following Emeljanov’s classification system [14,15], they were initially subdivided into three supertribes, currently upgraded to subfamilies by Bartlett et al. [16], and 12 tribes: Achilinae Stål, 1866 (Achilini Stål, 1866, Achillini Emeljanov, 1991), Apatesoninae Metcalf, 1938 (Apatesonini Metcalf, 1938, Ilvini Emeljanov, 1991, Seviini Emeljanov, 1991, Tropiphlepsiini Emeljanov, 1991), and Myconinae Fennah, 1950 (Amphignomini Emeljanov, 1991, Mycarini Emeljanov, 1991, Myconini Fennah, 1950, Plectoderini Fennah, 1950, Rhotalini Fennah, 1950, and the fossil tribe Waghildini Szwedo, 2006). The placement of another fossil tribe, Ptychoptilini Emeljanov, 1990, within the Achilidae or Derbidae, remains uncertain [5,17].

Achilidae are distributed worldwide, with a latitudinal profile particularly well represented in the northern hemisphere between 5° and 55° [13]. As obligate phytophagous species, they are predominantly associated with Fagales, Pinales, Ericales, and Rosales, accounting for 23.5%, 14.1%, 11.8%, and 8.2% of the records, respectively [13]. They are generally recognized as one of the planthopper groups best adapted to temperate climates [18].

The documented fossil taxa of Achilidae in the Cretaceous period are rare, with only three genera and four species recognized. Hamilton [19] described the first fossil genus *Acixiites* Hamilton, 1990, with *A. immodesta* Hamilton, 1990 (the type species), and *A. costalis* Hamilton, 1990, from the Crato Formation in Brazil representing the oldest fossil records of Achilidae from the Cretaceous period. Cockerell [20] initially described the first Myanmar amber planthopper taxon in the Delphacid genus *Liburnia* Stål, 1866, transferred to Achilidae by Shcherbakov [21], and redescribed by Szwedo [4] in the new genus *Niryasaburnia* Szwedo, 2004, with the type species *N. burmitina* (Cockerell, 1917). According to Cruickshank and Ko [22], the specimen belongs to the ‘old mines’ location in Hukawng Valley near Tanai. Brysz et al. [8] described the second genus from the Hukawng Valley also, *Amphignokachinia* Brysz & Szwedo, 2023, with the type species *A. subversa* Brysz & Szwedo, 2023, as the first mid-Cretaceous representative of the tribe Amphignomini in the subfamily Myconinae.

In this paper, a new Burmese amber species *Niryasaburnia nigrutomia* sp. nov. is described of Achilidae from the Kachin state (Tanai) of northern Myanmar, easily distinguished by its forewing pattern coloration and metatibiotarsal formula from *N. burmitina* (Cockerell, 1917). Additionally, a new Burmese amber genus *Sinuovenaxius* gen. nov., along with *S. kachinensis* sp. nov., is also described from this location. This discovery marks the third amber genus and the sixth fossil species of the family from the Cretaceous period. Furthermore, a new tribe Niryasaburniini trib. nov. is established within Myconinae to include the genera *Niryasaburnia* and *Sinuovenaxius* gen. nov. 

## 2. Materials and Methods

The specimens of new species originate from the Hukawng Valley (Tanai) of northern Myanmar and are now deposited in the College of Life Sciences, China West Normal University, Nanchong, Sichuan Province, China. To avoid any confusion and misunderstanding, all authors declare that the specimens in this study were not involved in armed and ethnic conflicts in Myanmar. Radiometric U-Pb zircon dating provided an accurate age of 98.79 ± 0.62 Ma of the deposit [23] which refers to the Cenomanian period of the mid-Cretaceous.

The amber polishing process involved wrapping and rubbing the specimens with a wet cloth using compound abrasive paste, followed by a final cleaning with water. Observations were performed using an Olympus SZX7 stereomicroscope (Olympus Corporation, Tokyo, Japan) and photos were captured using a Leica M205FA stereomicroscope (Leica Microsystems, Heerbrugg, Switzerland) equipped with a Leica MC190 HD camera (Leica Microsystems, Heerbrugg, Switzerland), and then automatically refined using the LAS X software version 2017.2.0 on a computer connected to the camera. Line drawings were created with CorelDRAW 2019 and SAI2.

The terminologies adopted for the forewing and hindwing venation follow, respectively, Bourgoin et al. [24], Bucher et al. [3] and Luo et al. [6], and for the male genitalia follows Bourgoin [25]. The metatibiotarsal formula (s-t)/tI/tII corresponds to the number of lateral spines (s) on the metatibia, the number of apical teeth (t) on the metatibia, the number of apical teeth (tI) on metatarsomere I, and the number of apical teeth (tII) on metatarsomere II. 

## 3. Systematic Paleontology

Order: Hemiptera Linnaeus, 1758

Suborder: Fulgoromorpha Evans, 1946

Superfamily: Fulgoroidea Latreille, 1807

Family: Achilidae Stål, 1866

Subfamily: Myconinae Fennah, 1950

 

Key to tribes of Myconinae

 

1. Metatibia with more than 4 lateral spines..................................................................................2
-. Metatibia with no more than 3 lateral spines.............................................................................3
2. CuA 2-branched in prenodal region on forewing; apical teeth of metatarsomeres I and II both with subapical platellar sensilla....................................................Waghildini Szwedo, 2006
-. CuA 3-branched in prenodal region on forewing; apical teeth of metatarsomere I without subapical platellar sensilla, but metatarsomere II with this sensilla..........................................................................................................Rhotalini Fennah, 1950
3. Genae with subantennal carinae; mesonotum without lateral carinae...........................................................................................Amphignomini Emeljanov, 1991
-. Genae without subantennal carinae; mesonotum with lateral carinae....................................4
4. MP with at least 6 terminals, CuA with at least 4 terminals on forewing......................................................................................................Myconini Fennah, 1950
-. MP with no more than 5 terminals, CuA with no more than 3 terminals on forewing.............................................................................................................................................5
5. Head with compound eyes around 2/3 length of pronotum.................................................................................................Plectoderini Fennah, 1950
-. Head with compound eyes around half length of pronotum..................................................6
6. Forewing with ScP+R and CuA forking before level of fusion of Pcu and A_1_; hindwing with CuA 3-branched............................................................................Mycarini Emeljanov, 1991
-. Forewing with ScP+R and CuA forking after level of fusion of Pcu and A_1_; hindwing with CuA 2-branched......................................................................................Niryasaburniini trib. nov.

 

Niryasaburniini Wang & Bourgoin trib. nov.

LSIDurn:lsid:zoobank.org:act:28F1995F-0AF4-49B3-BBAF-238A49F7533C

Type genus. *Niryasaburnia* Szwedo, 2004.

Composition. *Niryasaburnia* Szwedo, 2004, *Sinuovenaxius* gen. nov.

 

**Diagnosis:** Head with compound eyes around half length of pronotum. Vertex with anterior margin almost straight. Frons with median carina elevated; lateral margins subparallel above level of compound eyes, then strongly diverging below lower margin of compound eyes and curved. On forewings, basal cell narrow, elongated; precostal and stigmal areas without veinlets connecting anterior margin, no pterostigma area individualized; rather simple venation, MP with two or three terminals; CuA with two terminals; ScP+R and CuA forking late, well after the level of fusion of Pcu and A_1_; MP forking late well after nodal line level. On hindwings, RP simple with one terminal; both MP and CuA with two terminals; A_2_ simple, reaching posterior margin. Hind tibia with two or three small lateral spines including subgenual one; metatibial apical teeth strong, long, in row widely diverging apically; apical teeth of metatarsomeres I and II both with subapical platellar sensilla.

 

*Niryasaburnia* Szwedo, 2004

Type species: *Niryasaburnia burmitina* (Cockerell, 1917), by monotypy [4].

 

**Modified Diagnosis:** *Niryasaburnia* can be distinguished by the late forking of MP well after nodal line level, ScP+R and CuA forking after level of the fusion of Pcu+A_1_, ScP+R forking before end of clavus and before forking of CuA, C4 shorter than C3, RP 2-branched, MP forked twice with three terminals on forewing; the ventral margin of frons strongly diverging ventrally (twice as wide than between compound eyes) well below the lower margin of the compound eyes, frons with distinct median carina from anterior margin to posterior margin; long anteclypeus reaching the base of prolegs; rostrum exceeding metatrochanter; hind tibia with two lateral spines including subgenual one; metatibial apical teeth strong, long in row widely diverging apically, the apical teeth of metatarsomeres I and II both with subapical platellar sensilla.

 

*Niryasaburnia nigrutomia* Deng & Bourgoin sp. nov.

LSIDurn:lsid:zoobank.org:act:F17983FC-AEBF-4821-AC96-147897DCCE82

 

**Figure 1:**
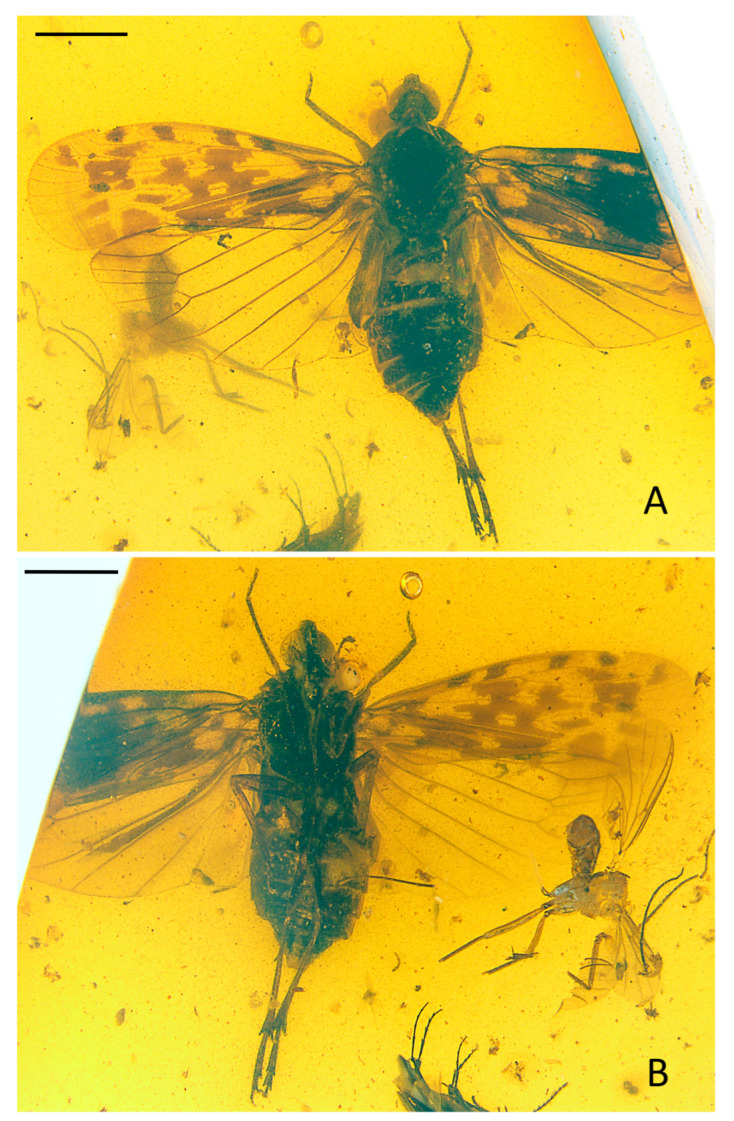
*Niryasaburnia nigrutomia* sp. nov., Holotype. (**A**) Adult, dorsal view; (**B**) adult, ventral view. Scale bar: 1 mm.

**Diagnosis:** This new species differs from *Niryasaburnia burmitina* (Cockerell, 1917) by the following characteristics: (1) tegmen with irregular brown patches as in Figure 1A (without patches in *N. burmitina*); (2) forewing, RA_2_ with only one terminal (two in *N. burmitina*); (3) transverse veins *r-m_1_* and *m-cu_1_* placed at same level of the forking of MP (distad in *N. burmitina*); (4) lateral carinae of the frontoclypeus without distinct granulation (present in *N. burmitina*); (5) metatibiotarsal formula: 2-8/8/7, versus 2-9/10/8 in *N. burmitina*.

**Etymology:** The name refers to the black thorax, *nigrum pronotum* in Latin, arbitrarily concatenated into *nigrutomia*.

**Type material:** Holotype, MDHP130. Female adult, in Burmese amber, from Hukawng Valley (Tanai location), Kachin State, Northern Myanmar. 

**Description:**

**Figure 2:**
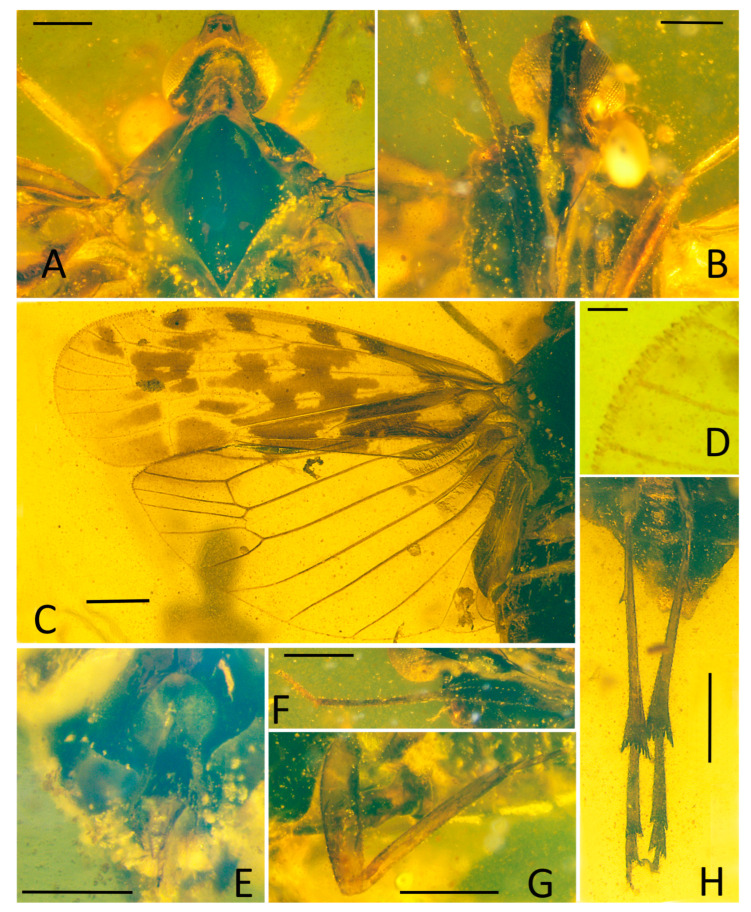
*Niryasaburnia nigrutomia* sp. nov., Holotype. (**A**) Head and thorax, dorsal view; (**B**) head, ventral view; (**C**) forewing and hindwing; (**D**) costal membrane of forewing; (**E**) female genitalia, dorsal view; (**F**) fore leg; (**G**) middle leg; (**H**) hind tibia and tarsus. Scale bar: 0.1 mm in (**D**), 0.3 mm in (**A**,**B**), 0.2 mm in (**E**), 0.5mm in (**C**,**F**–**H**).

**Figure 3:**
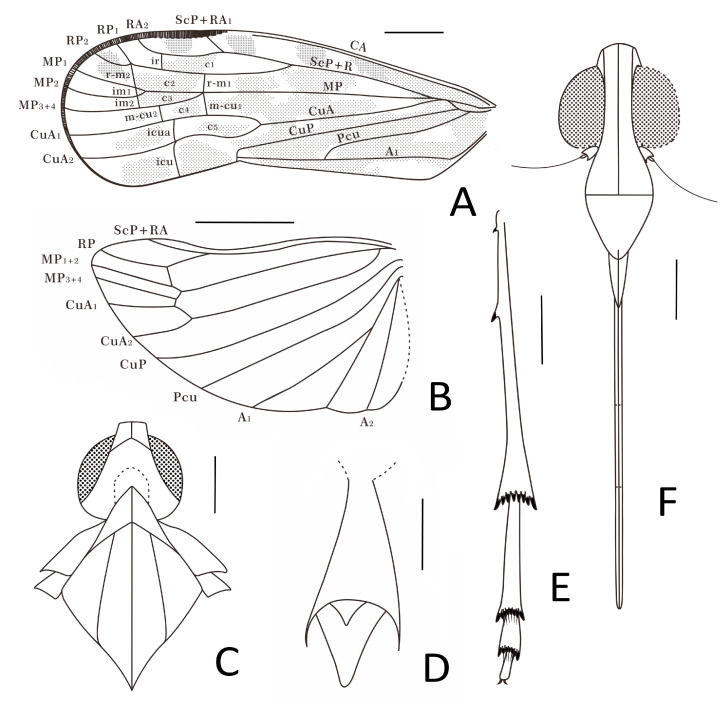
*Niryasaburnia nigrutomia* sp. nov., Holotype. (**A**) Forewing venation; (**B**) hindwing venation; (**C**) head and thorax, dorsal view; (**D**) female anal tube; (**E**) hind tibia and tarsus; (**F**) head, ventral view. Scale bar: 0.1 mm in (**D**), 0.3 mm in (**C**,**E**,**F**), 0.5 mm in (**A**), 1 mm in (**B**).

Small-size insect (Figure 1, Figure 2 and Figure 3). Total length including tegmina 4.99 mm. The forewing and hindwing in right side not completed due to the fossil conditions. Anal lobe of forewing in left side folded but well visible in right side. Head slightly split from thorax in the specimen. Frons and clypeus slightly slanted in ventral view in the specimen. The apex of left middle leg missing.

**Head.** Head width with compound eyes (Figure 2A) 0.61 mm. Vertex (Figure 2A) length 0.16 mm in midline, width in anterior margin 0.16 mm, width at middle 0.19 mm, width at base 0.26 mm. Compound eyes (Figure 2A) length 0.35 mm, width 0.12 mm. Frons (Figure 2B) length 0.65 mm, width in anterior margin 0.15 mm, width at middle 0.13 mm, width at base 0.31 mm. Clypeus (Figure 2B) length 0.37 mm. Rostrum (Figure 1B) 1.34 mm.

**Thorax.** Pronotum (Figure 2A) length 0.22 mm, width 1.04 mm. Mesonotum (Figure 2A) length 0.98 mm, widest width 0.90 mm.

**Forewings.** Tegmen (Figure 1A,B, Figure 2C and Figure 3A) length 3.96 mm in longest part, width 1.33 mm in widest part; RA_2_ with one terminal; transverse veins *r-m_1_* and *m-cu_1_* placed at same level of the forking of MP; tegmen hyaline, large area of irregular brown patches on tegmen, five of them almost equidistant located anteriorly on costal and radial area, wider markings posteriorly more or less confluent and irregular, as figured; clavus with darker brown markings; brown patches distally lighter or absent; costal membrane (Figure 2D) clearly visible.

**Hindwings.** Hindwing (Figure 1A and Figure 2C) 3.30 mm long in longest part, 1.78 mm in widest part.

**Legs.** Fore femur, tibia, and tarsus (Figure 1B and Figure 2F) length 0.79 mm, 0.84 mm, 0.36 mm; middle femur, tibia and tarsus (Figure 1B and Figure 2G) length 0.88 mm, 1.18 mm, 0.22 mm; hind femur, tibia and tarsus (Figure 1B and Figure 2H) length 0.82 mm, 1.34 mm, 0.75 mm; basitarsomere (Figure 2H) length 0.49 mm, metatarsomere II (Figure 2H) length 0.16 mm, metatarsomere III (Figure 2H) length 0.10 mm; metatibiotarsal formula: 2-8/8/7 (Figure 2H and Figure 3E).

**Female terminalia.** A female specimen but not clear enough and complete for description. In dorsal view, anal tube (Figure 2E and Figure 3D) sub-quadrangular with apical margin concave at middle, anal styles (Figure 2E and Figure 3D) developed.

 

*Sinuovenaxius* Wang & Bourgoin gen. nov.

LSIDurn:lsid:zoobank.org:act:7CAD226A-27DA-4C78-AFC8-CF6FEA73562C

Type species: *Sinuovenaxius kachinensis* sp. nov.

 

**Diagnosis:** From other Burmese amber Achilid fossils, the new genus approaches *Niryasaburnia*, but differs in the following characters on the forewing: (1) ScP+R forking late after end of clavus (before in *Niryasaburnia*), and after forking of CuA (before in *Niryasaburnia*); (2) all branches single after nodal line level (RP and MP_1+2_ forked once in *Niryasaburnia*); (3) CuA_2_ strongly sinuated at base (slightly sinuated in *Niryasaburnia*).

*Sinuovenaxius* gen. nov. differs from all currently known Achilids by its rather simple venation, by the combination of the following characters: (1) late forking of ScP+R after nodal level and after CuA forking; (2) as in several Plectoderini genera CuA_2_ on forewing distinctly sinuated but stronger in *Sinuovenaxius* with a late forking of CuA well after the level of fusion of Pcu +A_1._

**Etymology:** The Latin name refers to the sinuate CuA_2_ vein on the forewing, *sinuosus vena*, arbitrarily concatenated with -*xius*, gender masculine.

**Description:**

**Figure 4:**
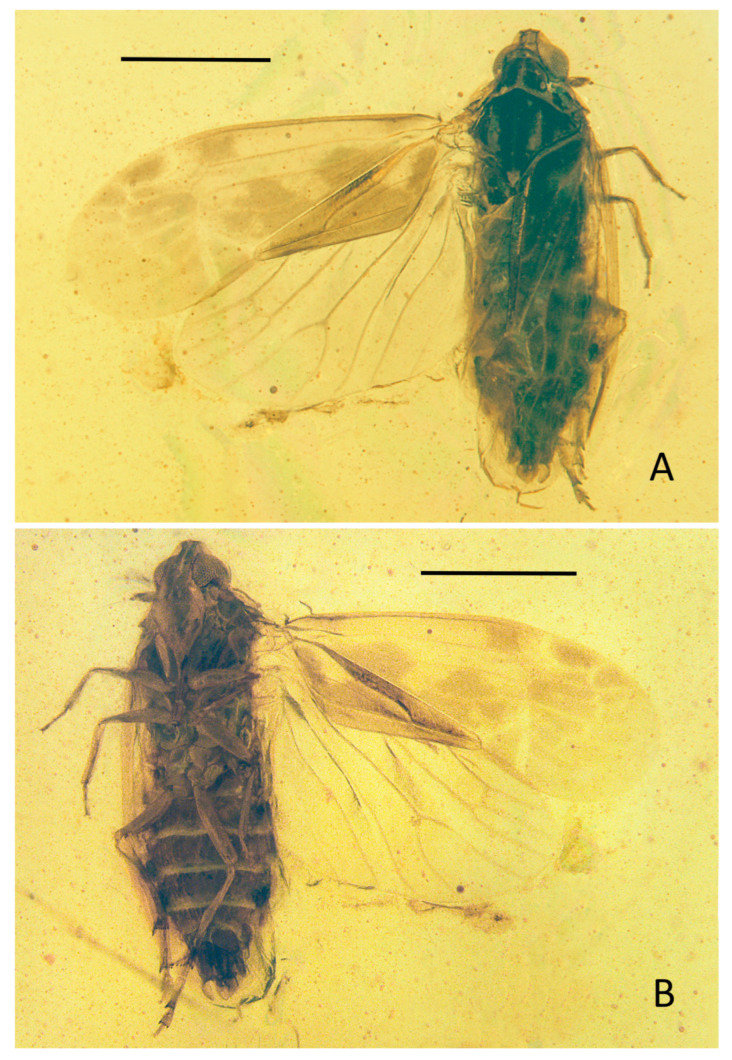
*Sinuovenaxius kachinensis* gen. et sp. nov., Holotype. (**A**) Adult, dorsal view; (**B**) adult, ventral view. Scale bar: 1 mm.

**Figure 5:**
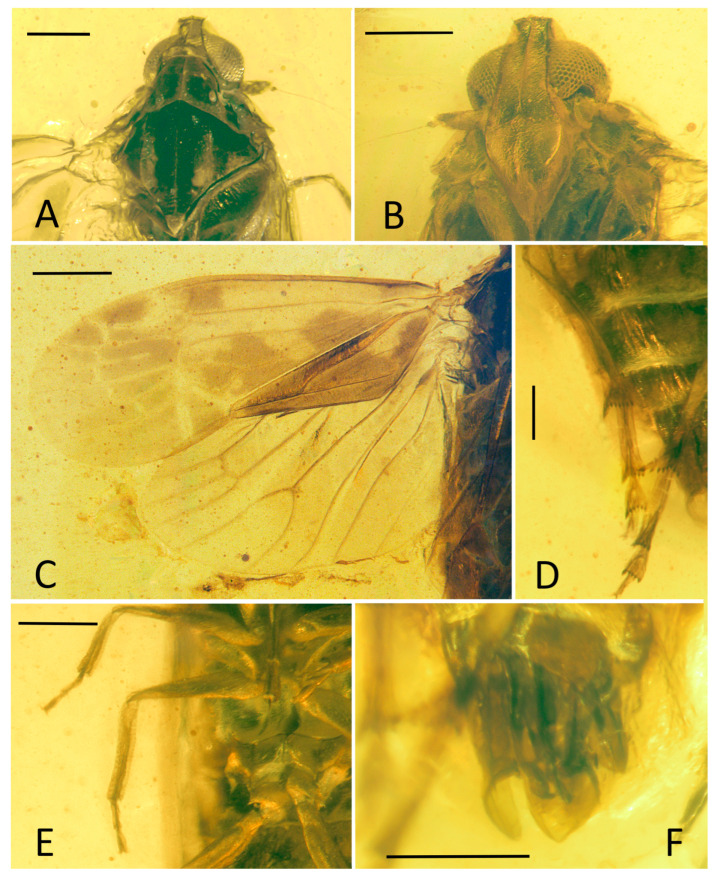
*Sinuovenaxius kachinensis* gen. et sp. nov., Holotype. (**A**) Head and thorax, dorsal view; (**B**) head, ventral view; (**C**) forewing and hindwing; (**D**) hind tibia and tarsus; (**E**) fore and middle legs; (**F**) male terminalia, ventral view. Scale bar: 0.3 mm in (**A**,**B**,**E**,**F**), 0.5 mm in (**C**), 0.2 mm in (**D**).

**Figure 6:**
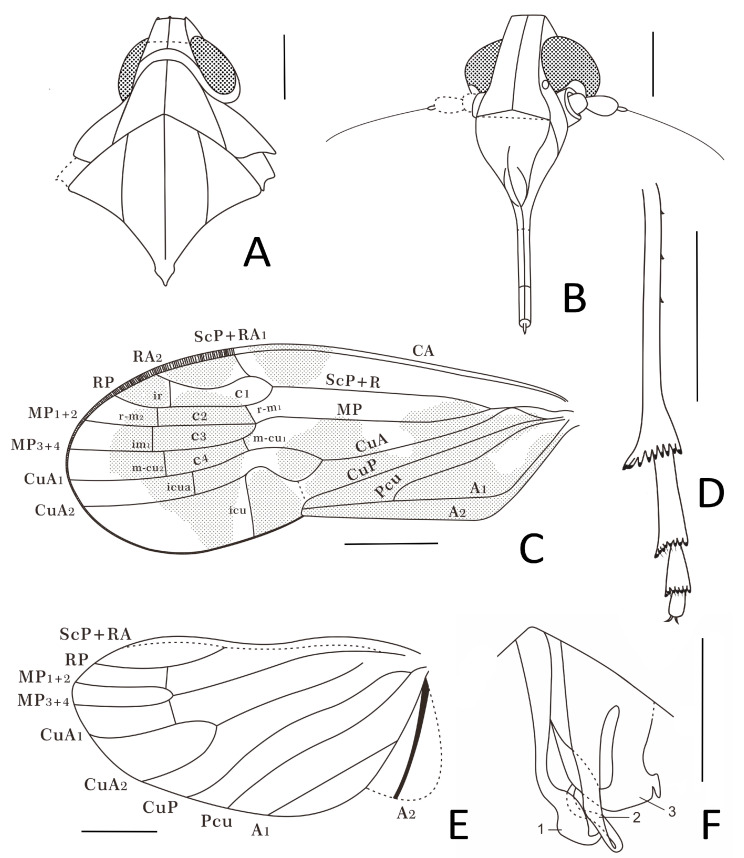
*Sinuovenaxius kachinensis* gen. et sp. nov., Holotype. (**A**) Head and thorax, dorsal view; (**B**) head, ventral view; (**C**) forewing venation; (**D**) hind tibia and tarsus; (**E**) hindwing venation; (**F**) processes of phallic complex, ventral view. Scale bar: 0.3 mm in (**A**,**B**), 0.5 mm in (**C**–**E**), 0.2 mm in (**F**).

**Head.** Vertex (Figure 4A, Figure 5A and Figure 6A) trapezoid, slightly wider than long, with a distinct median carina, reaching anterior and posterior margins of vertex; anterior margin almost straight, surpassing the upper margin of compound eyes; lateral margins inclined outwards to the base; posterior margin roundly concave. Frons (Figure 4B, Figure 5B and Figure 6B) much longer than wide, the length in midline of frons about 2.3 times longer than wide, with distinct elevated median carina from the apical margin to the base of frons; apical margin straight; lateral margins subparallel above the lower margin of compound eyes, and then expanded outward. Median ocellus absent. Gena (Figure 5B and Figure 6B) with a pair of lateral ocelli touching compound eyes. Compound eyes (Figure 5B and Figure 6B) large, bulged. Antennae (Figure 5B and Figure 6B) with scape and elongated pedicel globulous; in frontal view, pedicel surpassing external margin of compound eyes; flagellum twice longer than pedicel, basal bulb of flagellum oval. Frontoclypeal suture (Figure 5B and Figure 6B) slightly angular. Clypeus (Figure 5B and Figure 6B) large, dorsally almost same as frons at length, triangular; with median carina well present in the basal 2/3; lateral margins converging to base. Rostrum (Figure 4B, Figure 5E and Figure 6B) short, just reaching mesocoxae.

**Thorax.** Pronotum (Figure 5A and Figure 6A) strong, wide and large, length along midline longer than length of vertex, saddle-like; anterior margin roundly convex, reaching to the middle level of compound eyes; posterior margin angularly concave, forming an angle around 120° at middle; disc with three distinctly elevated carinae derived from anterior margin reaching to posterior margin. Mesonotum (Figure 5A and Figure 6A) diamond-shaped, slightly wider than length in midline, about 3.3 times longer in midline than the length of pronotum in midline; with three obvious longitudinal carinae from anterior margin to posterior margin, lateral carinae subparallel to median carina, the lateral areas slant ventrally. Mesoscutellum (Figure 5A and Figure 6A) with lateral margins subparallel, and then triangular. 

**Forewings.** Tegmen (Figure 4A, Figure 4B, Figure 5C and Figure 6C) distinctly longer than wide, color markings interspersed on the surface, with a subapical line delimitating seven distal apical open cells. Costal margin slightly curved; apical forewing margin rounded. Clavus almost half of tegmen length, Pcu and A_1_ fused slightly after middle of clavus, clavus closed, the stem of Pcu+A_1_ reaching CuP at the apex of clavus. The common stalk ScP+R very long, subparallel to costal margin; ScP+R forking close to nodal line level after apex of clavus level, at about 2/3 of tegmen length; ScP+RA_1_ single, reaching anterior margin; RA_2_ and RP base curved just distal to fork, both with only one terminal. Common stem ScP+R+MP short; MP bifurcated in two branches MP_1+2_ and MP_3+4_ around apical 1/3 of tegmen, slightly after the forking of ScP+R, both MP_1+2_ and MP_3+4_ simple and nearly straight. Two *r-m*, respectively, after the ScP+R fork and *ir*; *im_1_* slightly after *r-m_2_*; two *m-cu*, respectively, after proximally stalked C3 and before *im_1_*. C1 and C5 expanded at base; C2 and C3 subparallel; C4 quadrangular, shorter than C3. Stem CuA bifurcated in CuA_1_ and CuA_2_ slightly before apex of clavus; CuA_1_ single, distinctly curved upward in the basal half; CuA_2_ single, strongly S-shaped sinuated proximally; *icua* before other subdistal veinlets, but C5 still the longest cell; *icu* long, from the middle of C5 to forewing margin.

**Hindwings.** Hindwing (Figure 4A, Figure 4B, Figure 5C and Figure 6E) more acute at MP_1+2_ level; venation pattern similar to *Niryasaburnia*. ScP+RA and RP separating at 3/5 length of costal margin; RP simple, reaching apical margin; MP bifurcate to simple MP_1+2_ and MP_3+4_ distad to the forking of ScP+RA and RP; CuA with two terminals, fork of CuA nearly at the same level as the fork of ScP+RA and RP; veins CuP and Pcu simple, slightly sinuate; A_1_ straight; A_2_ thick, slightly arcuate anteriorly, reaching posterior margin; transverse veins *r-m* and *m-cu* slightly after the forking of MP.

**Legs.** Fore and middle legs (Figure 4B and Figure 5E) robust; hind femur (Figure 4B) equal width, tibia (Figure 5D and Figure 6D) with three lateral spines in basal half including subgenual one, apical portion slightly wider with strong apical teeth placed in a row strongly widening; basitarsomere (Figure 5D and Figure 6D) longer than the combined length of metatarsomeres II and III (Figure 5D and Figure 6D), apical teeth on metatarsomeres I and II equal length, subapical platellar sensilla present.

 

*Sinuovenaxius kachinensis* Wang & Bourgoin sp. nov.

LSIDurn:lsid:zoobank.org:act:1C4A45C9-8067-4463-A8CB-93EB726978C8

 

**Diagnosis:** This species could be quickly recognized by the processes of the phallic complex and the metatibiotarsal formula 3-9/8/7.

**Etymology:** This name refers to the location of this species in Kachin state from Myanmar.

**Type material:** Holotype, MDHP78. Male adult, in Burmese amber, from Hukawng Valley (Tanai location), Kachin State, Northern Myanmar.

**Description:**

Small-size insect (Figure 4, Figure 5 and Figure 6), well preserved. Total length including tegmina 3.09 mm. 

**Head.** Head width with compound eyes (Figure 5A) 0.49 mm. Vertex (Figure 5A) length 0.12 mm in midline, width in anterior margin 0.13 mm, width at middle 0.16 mm, width at base 0.20 mm. Compound eyes (Figure 5A) length 0.27 mm, width 0.16 mm. Antennae (Figure 5B) scape length 0.07 mm, pedicel length 0.18 mm, flagellum length 0.43 mm. Frons (Figure 5B) length 0.42 mm, width in anterior margin 0.13 mm, width at middle 0.18 mm, width at base 0.28 mm. Clypeus (Figure 5B) length 0.40 mm. Rostrum (Figure 4B) 0.39 mm. 

**Thorax.** Pronotum (Figure 5A) length 0.20 mm, width 0.84 mm. Mesonotum (Figure 5A) length 0.66 mm, widest width 0.74 mm. 

**Forewings**. Tegmen length 2.72 mm in longest part, width 1.06 mm in widest part; tegmen (Figure 4A,B and Figure 5C) hyaline, the surface of tegmina with irregular brown patches on apical half in the cell areas, beyond ScP, on basal 1/4 and the middle area from MP to clavus margin; the markings close to clavus darker; veinlets (Figure 5C) in apical half and CuP yellow, others brown.

**Hindwings.** Hindwing (Figure 4A,B and Figure 5C) hyaline, 2.24 mm long in longest part, 1.22 mm in widest part.

**Legs.** Fore femur, tibia, and tarsus (Figure 4B and Figure 5E) length 0.56 mm, 0.37 mm, 0.22 mm; middle femur, tibia, and tarsus (Figure 4B and Figure 5E) length 0.55 mm, 0.46 mm, 0.23 mm; hind femur, tibia, and tarsus (Figure 4B) length 0.55 mm, 0.68 mm, 0.51 mm; basitarsomere (Figure 5D) length 0.27 mm, metatarsomere II (Figure 5D) length 0.12 mm, metatarsomere III (Figure 5D) length 0.12 mm; metatibiotarsal formula: 3-9/8/7 (Figure 5D and Figure 6D).

**Male terminalia.** In ventral view, phallic complex (Figure 5F and Figure 6F) asymmetric; the left process of periandrium (1) long, with apex broader and sinuated, curved to middle; the middle process, aedeagus *s.s.* (2), slender and twisty to left side in the apex; the right process of periandrium (3) shorter and broad, with a large sharp triangular process in the left side directed to basal and a small short spine-like process in the right side directed to caudal.

## 4. Discussion

According to Emeljanov’s key [14] to suprageneric taxa, Myconinae are characterized by the contiguous base of MP with ScP+R on hindwings, with the second anal vein A_2_ simple (as opposed to being branched in Achilinae), A_2_ mostly not reaching wing margin. Using the key, the taxon fits superficially into couplet 8(7) [14], which corresponds to Mycarini: hindtibia usually with three lateral spines, rarely two, subgenual spine always present, and MP with no more than four terminals on forewing. However, (1) the forking of ScP+R and CuA on the forewing basad of fusion of Pcu and A_1_ (obviously distad in Niryasaburniini), (2) MP usually with four terminals on tegmen (with two or three terminals in Niryasaburniini), and (3) CuA 3-branched on hindwing (2-branched in Niryasaburniini), make it easily separate to Niryasaburniini trib. nov. This tribe is also close to Breddiniolini Fennah, 1950 [14], which possesses three lateral spines on the metatibia and hindwing A_2_ reaching to the posterior margin. However, it differs from this current Derbidae taxon [5] by its simpler venation pattern. Niryasaburniini is also distinct from Plectoderini, by the vertex with compound eyes around half length of the pronotum (compared to 2/3 length in Plectoderini [26]), the anterior margin of the vertex is straight (usually rounded or angulately protruded in Plectoderini [26]), hindwing with a 2-branched CuA and the A_2_ reaching the posterior margin (3-branched CuA and A_2_ not reaching in Plectoderini), and one subgenual metatibial spine (lacking proximal subgenual spine in Plectoderini [14]). With all Plectoderini, Niryasaburniini trib. nov. shares a 2-branched CuA on forewing with two simple long branches. 

Myconinae has previously been reported from the Cretaceous, notably with the tribe Amphignomini represented by the genus *Amphignokachinia* in Burmese amber. However, its mesonotum without lateral carinae, the forewing with marked pterostigma, CuA_1_ branched, hindwing CuA 3-branched, genae with subantennal carinae, and metatarsomeres without subapical platellar sensilla [8] allow to quickly distinguish it from the new tribe Niryasaburniini trib. nov. 

Both *Niryasaburnia* and the new genus *Sinuovenaxius* gen. nov. share the same peculiar hindwing patterns: simple RP, both MP and CuA with two terminals and simple A_2_. They also share their head capsule conformation with compound eyes half length of the pronotum, anterior margin of vertex almost straight; also, both exhibit a late forking of ScP+R and CuA, MP with two or three terminals and a 2-branched CuA on forewing; two or three metatibial lateral spines and the apical teeth of metatarsomeres I and II both with subapical platellar sensilla. Altogether these characters allow to separate them from all the known tribes of Achilidae into a new tribe. Future phylogenetic analysis will be needed to confirm if this new taxonomic grouping reflects a valid phylogenetic clade.

## 5. Conclusions

A new Achilidae tribe, Niryasaburniini trib. nov., in the subfamily Myconinae is proposed to accommodate two mid-Cretaceous Burmese amber genera: *Niryasaburnia*, including a second new species, and a newly described genus *Sinuovenaxius* gen. nov. 

Several Achilidae taxa from Burmese amber await formal description and placement within the current classification, posing a taxonomic challenge. Refinement is needed, particularly through robust molecular phylogeny analyses. The ongoing discovery of new taxa within the planthopper fauna of Burmese amber illustrates the richness and diversity of the taxon during the Cretaceous, likely also boosted by the insular condition of the biotope, favoring endemism and speciation events [27].

## Data Availability

All relevant data are available from the text and figures.
